# *Bordetella pertussis* Isolates from Argentinean Whooping Cough Patients Display Enhanced Biofilm Formation Capacity Compared to Tohama I Reference Strain

**DOI:** 10.3389/fmicb.2015.01352

**Published:** 2015-12-08

**Authors:** Laura Arnal, Tom Grunert, Natalia Cattelan, Daan de Gouw, María I. Villalba, Diego O. Serra, Frits R. Mooi, Monika Ehling-Schulz, Osvaldo M. Yantorno

**Affiliations:** ^1^CINDEFI–Centro Científico Tecnológico CONICET La Plata, Facultad de Ciencias Exactas, Universidad Nacional de La Plata Buenos Aires, Argentina; ^2^Functional Microbiology, Institute of Microbiology, Department of Pathobiology, University of Veterinary Medicine ViennaVienna, Austria; ^3^Laboratory of Pediatric Infectious Diseases, Department of Pediatrics, Radboud University Medical CentreNijmegen, Netherlands; ^4^Laboratory of Medical Immunology, Department of Laboratory Medicine, Radboud University Medical CentreNijmegen, Netherlands; ^5^Mikrobiologie, Institut for Biologie, Humboldt-Universitat zu BerlinBerlin, Germany; ^6^Netherlands Centre for Infectious Disease Control, National Institute for Public Health and the EnvironmentBilthoven, Netherlands

**Keywords:** whooping cough, *Bordetella pertussis*, clinical isolates, biofilm, proteomic, real time PCR

## Abstract

Pertussis is a highly contagious disease mainly caused by *Bordetella pertussis*. Despite the massive use of vaccines, since the 1950s the disease has become re-emergent in 2000 with a shift in incidence from infants to adolescents and adults. Clearly, the efficacy of current cellular or acellular vaccines, formulated from bacteria grown in stirred bioreactors is limited, presenting a challenge for future vaccine development. For gaining insights into the role of *B. pertussis* biofilm development for host colonization and persistence within the host, we examined the biofilm forming capacity of eight argentinean clinical isolates recovered from 2001 to 2007. All clinical isolates showed an enhanced potential for biofilm formation compared to the reference strain Tohama I. We further selected the clinical isolate *B. pertussis* 2723, exhibiting the highest biofilm biomass production, for quantitative proteomic profiling by means of two-dimensional fluorescence difference gel electrophoresis (2D-DIGE) coupled with mass spectrometry, which was accompanied by targeted transcriptional analysis. Results revealed an elevated expression of several virulence factors, including adhesins involved in biofilm development. In addition, we observed a higher expression of energy metabolism enzymes in the clinical isolate compared to the Tohama I strain. Furthermore, all clinical isolates carried a polymorphism in the *bvgS* gene. This mutation was associated to an increased sensitivity to modulation and a faster rate of adhesion to abiotic surfaces. Thus, the phenotypic biofilm characteristics shown by the clinical isolates might represent an important, hitherto underestimated, adaptive strategy for host colonization and long time persistence within the host.

## Introduction

*Bordetella pertussis* is a human-restricted pathogen specifically adapted to infect the respiratory tract producing whooping cough or pertussis. Despite the success of mass immunization in reducing the incidence of the disease in the 1950s, after six decades of sustained high vaccination coverage, whooping cough remains endemic with epidemic cycles every 2–5 years (Mooi et al., 1998, 2001, 2009, 2014; Heikkinen et al., 2007; King et al., 2008). Although the disease has been associated to an acute infection, mainly affecting unvaccinated infants aged <6 months, in the last two decades, a shift in the incidence toward vaccinated children, adolescents, and adults has become increasingly evident (Cherry, 1999; Hellenbrand et al., 2009; de Greeff et al., 2010). This new scenario represents a significant health concern, since these individuals could provide reservoirs for *B. pertussis* transmission. Several reasons for the resurgence and persistence of pertussis in the population have been discussed, including: waning immunity over time, variation between circulating isolates and vaccine strains as a result of constant pathogen adaptation, and reduced efficiency of vaccine formulations (He et al., 2003; Hewlett and Edwards, 2005; Brinig et al., 2006; Bottero et al., 2007; Berbers et al., 2009; Elomaa et al., 2009; Lavine et al., 2011). For the commercial production of both, cellular and acellular vaccines, *B. pertussis* cells are grown in stirred bioreactor operated in batch culture. This planktonic (free-floating) mode of growth does not reflect the lifestyle of the pathogen in its host, where bacteria must primarily adhere to ciliated respiratory epithelial cells; in this hostile environment, bacteria must resist mucociliary clearance and avoid the immune system’s mechanisms, adjusting their growth state and virulence accordingly to survive.

Numerous reports provide evidence that the ability of pathogens to adhere and grow attached to tissues’ surfaces in microbial communities, known as biofilm, is crucial for the development and progression of human infections (Costerton et al., 1999; Hall-Stoodley et al., 2004). Generally, biofilm development, which is often associated to an enhanced resistance to antimicrobial agents and host defenses, is considered as an important survival strategy for bacteria (Ito et al., 2009; Hoiby et al., 2010; Gurung et al., 2013). In addition, the switch from planktonic to biofilm lifestyle is accompanied by significant changes in bacterial metabolism and phenotypic features, which represent a unique challenge for the development of novel prophylactic therapeutics. We as well as others have shown the capacity of *Bordetella* spp. to grow adhered to abiotic and biotic surfaces as biofilms (Irie et al., 2004; Mishra et al., 2005; Serra et al., 2008, 2011). The two-component sensory transduction system BvgAS, which controls the expression of nearly all known virulence factors, was reported to play an important role in the regulation of biofilm formation for these bacteria (Irie et al., 2004; Mishra et al., 2005). However, the role of biofilm in *B. pertussis* pathogenesis is not yet fully understood and, with a few exceptions (de Gouw et al., 2014), this mode of growth is still largely ignored when new antigens are selected for the formulation of novel pertussis vaccines. Thus, the aim of this study was to compare the biofilm formation by a well-characterized reference strain and eight *B. pertussis* clinical isolates, retrieved from children with pertussis during a 7-years period in Argentina. A comparative analysis, employing proteomics, targeted transcriptomics, and complementary genetic studies including the reference strain Tohama I (which has been sub-cultured *in vitro* since the 1950s), and the clinical isolate Bp2723 (selected by its high capacity of biofilm growth) were carried out to gain insight into the mechanisms responsible for the different behavior of sessile cells exposed to similar external conditions. Our results support the hypothesis that the phenotypic heterogeneity between the reference strain and clinical isolates reflects a specific adaptation of clinical *B. pertussis* to its host. Interestingly, a single nucleotide polymorphism in the *bvgS* gene in all clinical isolates was found, which might implicate an intrinsic feature of the circulating cells. This mutation allowed a fast adaptive response of modulated cells (vir-), incubated under non-modulating conditions, to adhere on abiotic surfaces. Our results foster the hypothesis that these bacteria have developed a repertoire of mechanisms that enable adaptive response to growth adhered to surfaces, allowing these cells to persist in unfriendly environments.

## Materials and Methods

### Bacterial Strains and Culture Conditions

*Bordetella pertussis* Tohama I strain -isolated in Japan in the 1950s- was obtained from the Collection of Institute Pasteur, Paris, France (CIP 8132); BPSM, a streptomycin resistant (Smr) strain derivative from Tohama I; Bp_K705E_, a mutant derivative of BPSM in which the amino acid lysine (K) at position 705 of the BvgS has been replaced by glutamic acid (E) (Herrou et al., 2009); and eight *B. pertussis* clinical isolates collected at La Plata Children’s Hospital (Hospital Interzonal de Agudos Especializado en Pediatría Sor Maria Ludovica, La Plata, Argentina) from 2001 to 2007 (**Table 1**) were used throughout this study. Stock cultures were grown on Bordet–Gengou agar (Difco Laboratories, Detroit, MI, USA) plates supplemented with 1% w/v Bactopectone (Difco) and 15% v/v defibrinated sheep blood (Instituto Biológico, Ministerio de Salud, La Plata, Argentina; BGA) for 72 h at 37°C. Colonies were cultured for others 48 h and then inoculated into 250-mL Erlenmeyer flasks containing 50-mL of Stainer–Scholte (SS) broth and incubated for 24 h at 37°C on a rotatory shaker (160 rpm). The suspension was used as inoculum for 1-L Erlenmeyer flasks containing 200-mL of SS broth. The initial optical density at 650 nm (OD_650_) was adjusted to 0.2 and the flaks were incubated for 24 h with agitation. Bacteria were harvested (15°C, 8000 ×*g*, 20 min) at exponential phase, frozen using liquid Nitrogen for 30 s and stored for 48 h at –80°C before being freeze-dried. To study the growth kinetic in liquid medium, samples were collected every 2 h and the OD_650_ was measured. Three independent experiments were performed for each strain, averages and standard deviations of the experimental data obtained are indicated in the figures. Specific growth rates (μ) were obtained from curves ln OD_650_ vs. time.

**Table 1:** *Bordetella pertussis* reference strain and clinical isolates used in this study.

Strain	Year of isolation	Patient age (weeks)	Source	*PtxA*	*Fim*	*Prn*
Bp Tohama I	1954	–	Japan	2	2	1
Bp2723	2001	8	Argentina	1	3	1
Bp1918	2003	12	Argentina	1	3	2
Bp2930	2004	17	Argentina	1	3	2
Bp3495	2004	4	Argentina	1	3	2
Bp7470	2005	8	Argentina	1	3	2
Bp162	2006	8	Argentina	1	3	2
Bp492	2006	6	Argentina	1	3	2
Bp892	2007	12	Argentina	1	3	2
B_PK705E_	2009		Japan	2	2	1
BPSM	1994		Tohama derivative	2	2	1


### Biofilm Cultures

The biofilm growth was performed as indicated previously (de Gouw et al., 2014). Briefly, for each *B. pertussis* strain, a bacterial suspension of planktonic cells (24 h of growth), adjusted to an OD_650_ = 1.0 (1.0 × 10^9^ CFUs/mL) was incubated with 20 g of polypropylene beads (4.2 mm diameter and 2 mm high, average density: 0.901 g/L, Petroken SA, Argentina) contained in glass tubes for 4 h at 37°C under static conditions. The cell suspension was drained and 20-mL of fresh medium were added to each reactor (glass tubes). The tubes were incubated for 72 h on roller drums under 60 rpm agitation. The culture medium was replaced every 24 h by fresh broth. Before harvest, the beads were washed three times with phosphate buffer saline (PBS) and then used for crystal violet staining. Growth kinetic for sessile cells of *B. pertussis* 2723 strain was evaluated analyzing colony forming units (CFU) every 24 h until 120 h of development. These experiments were performed by triplicate. In a similar way, a semi-continuous biofilm culture was performed to obtain samples able to be analyzed by confocal laser scanning microscopy (CLSM). Duplicates of Bp Tohama I and Bp2723 biofilms were grown attached to glass cover slips. In a first step, bacteria coming from a 24 h planktonic culture were incubated during 4 h with the cover slips inside a Petri dish. Then, broth was changed for fresh medium and incubated under agitation (60 rpm) for 72 h. Every 24 h the medium was changed for fresh broth. After 72 h the cover slips were carefully washed with PBS and stained for CLSM analysis.

For proteomic studies sessile cells were cultured on polypropylene beads contained in column bioreactors as was previously described (Serra et al., 2008) with minor modifications. Briefly, *B. pertussis* Tohama I strain or the clinical isolate *B. pertussis* 2723 were grown in 200-mL SS broth for 24 h and then used to inoculate column reactors. After 4 h of static incubation to allow cell attachment, the suspension was drained to remove non-adhered cells and 200-mL of fresh SS broth were added to each column. Bioreactors were incubated with a constant air supply (0.1 vvm) at 37°C for 72 h (mature biofilm stage). Every 24 h the broth was replaced by fresh medium. Afterward, polypropylene beads were washed three times with PBS prior to harvest the cells. The biofilm was detached from the surface by lightly agitation on PBS, subsequently, cells were harvested (15°C, 8000 × *g*, 20 min) and immediately frozen in liquid Nitrogen and stored at –80°C before being freeze-dried.

### Fourier Transform Infrared Spectroscopy (FT-IR)

For infrared analysis of biofilms each strain was grown in 6-well plates. After 72 h incubation, the biofilms were washed three times with distilled water and the biomass attached to the wells were suspended in bi-distilled water, adjusting the OD_650_ to 10. Samples were prepared from three independent assays by triplicate in each case. One hundred microliters of each bacterial suspension were transferred to an optical cell of zinc selenide (ZnSe) and vacuum dried (3.6 kPa) to obtain transparent films on the cell. FT-IR absorption spectra from 4,000 to 600 cm^-1^ were acquired with a FT-IR spectrometer (Spectrum One, Perkin Elmer, USA) as was reported (Serra et al., 2007). Infrared analysis was carried out as by means of OPUS 7.0 software (Bruker Optics, USA).

### Quantification of Biofilm Biomass

Biofilm biomass was quantified using the crystal violet assay described by O’Toole and Kolter (1998) adapted to the system. The absorbance of the solubilized dye was measured at 590 nm (OD_590_). Triplicates were made for each strain and *t*-Student’s test was used to compare absorbance against *B. pertussis* Tohama I’s biofilm. Samples were considered significantly different when *p* ≤ 0.05. CLSM was used to study the architecture and quantitative information of 72 h biofilms. An inverted confocal microscope Carl Zeiss LSM510-Axiovert 100M (Germany) was used as previously reported (Serra et al., 2007). Briefly, biofilms coming from semi-continuous culture, adhered to glass cover slips were first washed very carefully in PBS, and fixed with 4% paraformaldheyde. Then, adhered cells were rinsed in PBS, stained for 20 min with Acridine Orange and washed three times. In order to obtain quantitative information of mature biofilm structure, images were analyzed by COMSTAT software (Heydorn et al., 2000).

### Preparation of Soluble Protein Fraction

Cytosolic proteins were obtained following the protocol described by Ehling-Schulz et al. (2002) with minor modifications. Planktonic and sessile freeze-dried bacteria were suspended in detergent buffer containing 7 M urea, 2 M thiourea, 4% CHAPS and 30 mM TRIS (Sigma, St. Louis, MO, USA), cooled and then passed through a precooled French pressure cell (SLM AMINCO) working at 140 mPa two times. Cellular debris was harvested by centrifugation (4°C, 10000 × *g*, 15 min). The supernatant was transferred to ultracentrifuge tubes (Beckman, USA) and centrifuged at 30000 × *g* for 40 min at 15°C. The supernatant containing cytosolic proteins was stored in aliquots at –80°C. Protein concentration was estimated using the 2-D Quant kit following the manufacture’s protocol (GE Healthcare, Amersham Biosciences).

### 2D-DIGE

For two-dimensional difference gel electrophoresis (2D-DIGE) protein samples were minimally labeled as previously described (Radwan et al., 2008) with minor modifications. CyDye DIGE^TM^ fluorescent dyes (GE Healthcare Life Science, Munich, Germany) were used to label 33 μg of proteins per sample using 8 nmol dye/mg proteins. For each mode of growth three biological replicates were used. Biofilm and planktonic samples from each strain were labeled with Cye3 and Cye5. The internal standard comprising a pool of equal amounts from all samples was labeled with Cye2. Isoelectric focusing was carried out on an IPGphor III (GE Healthcare, Amersham Biosciences) system using 18 cm IPG Dry strips with linear pH gradients of 4–7 and 6–9 (all GE Healthcare, Amersham Biosciences). The IPG strips were rehydrated over night with rehydration buffer [7 M urea, 2 M thiourea, 4% (w/v) CHAPS, 0.4% (w/v) DTT, 0.5% carrier ampholytes] at room temperature. DTT 0.4% (w/v) and carrier ampholytes 0.5% (v/v) were added to the mixed proteins samples in detergent buffer and the final volume was adjusted to 50 μL with rehydration buffer. Protein samples were then loaded onto the strips via loading cups. pH 4–7 strips were focused for a total of 36 KVh and pH 6–9 strips were focused for a total of 27 KVh. The IPG strips were reduced with 1% w/v DTT for 15 min and alkylated using 4% (w/v) iodoacetamide for 15 min in equilibration buffer (6 M urea, 30% (v/v) glycerin, 2% (w/v) SDS) and SDS-PAGE (12.5% T) was subsequently performed overnight (13 mA per gel) using an Ettan Dalt Six Electrophoresis Chamber (GE Healthcare, Amersham, Biosciences).

### Imaging and Data Processing

Fluorescence images of the gels were acquired using a Typhoon 9400 scanner (GE Healthcare). Data analysis was performed with the DeCyder software version 7.0 (GE Healthcare). Spot detection, matching, and normalization were performed using a standard algorithm of the software. Spot matching as well as spot quality of proteins of interest were manually checked eliminating false positives. To assess the reproducibility of gel replicates, principal component analysis (PCA) was carried out employing the DeCyder analysis module. For PCA all spots within the ANOVA 95th confidence interval were included. Spots showing more than threefold changes (*p* ≤ 0.05) in abundance between the strains growing as biofilm or in planktonic mode were considered as significant differences, manually excised from silvers stained gels (Blum et al., 1989; Miller and Gemeiner, 1992) and subjected to mass-spectrometry for protein identification.

### In-gel Trypsin Digestion and MS-Based Protein Identification

Protein identification was carried out using a matrix-assisted laser desorption/ionization time-of-flight (MALDI–TOF) mass spectrometer (Bruker Daltonics, Ultraflex I) in MS and MS/MS modes. Spot distaining, in gel digestion and sample purification using Zip-Tipμ-C18 (Millipore) pipette tips were performed as reported previously (Blum et al., 1989). Samples were applied on a disposable target plate (Bruker Daltonics, PAC target) pre-spotted with α-cyano-4-hydroxycinnamic acid as matrix. Spectral pre-processing and peak annotation were carried out using FlexAnalysis 3.0 and Biotools 3.2 (Bruker Daltonics). Processed MS and MS/MS spectra were submitted to MASCOT server (Matrix Science) searching the database NCBInr restricting to *B. pertussis* Tohama I strain. Peptide mass fingerprint (PMF) search parameters were set for mass accuracy: <150 ppm, fixed modification: carbamidomethylation, variable modifications: methionine oxidation and acetylation at the protein N-terminal end, and missed cleavages: one. Based on the measured PMF at least one peptide was selected for MS/MS experiments. Search parameters were identical to PMF experiments, except for product ion tolerance (±1.0 Da). A protein was considered as identified, if the scores of database searches clearly exceeded the algorithm’s significance threshold (*p* < 0.05) for PMF data and for sequence tag ion analyses of at least one peptide.

### RNA Isolation, cDNA Synthesis, and Quantitative Real-time PCR (qRT-PCR)

Total RNA was isolated from planktonic and biofilm bacteria using Trizol reagent (Life Technologies, Invitrogen) following manufacturer’s instructions. The RNA was treated with DNase I (Promega, Madison, WI, USA) to remove contaminating DNA and cDNA synthesis was performed using hexamers primers (Promega, Madison, WI, USA) and M-MLV retrotranscriptase enzyme (Invitrogen, Carlsbad, CA, USA) following supplier’s protocol. Specific primers were used to determine transcript levels of the selected genes (**Table 2**). SYBR premix (Thermo Scientific) was used for qPCR assays following manufacturer’s instructions. Reactions were carried out on triplicate samples, including technical duplicates. Relative mRNA expression ratios of selected genes were normalized to the expression of 16S rRNA. Calculations for comparison between samples were performed using the ΔΔ*CT* (where *CT* is threshold cycle) method as described by Conover et al. (2012). In the case of differences in primers efficiency a modification was done following the method described by Pfaﬄ (2001).

**Table 2:** List of primers used for amplification of virulence genes, *bvgA* and *bvgS* genes of *B. pertussis* reference strain and the clinical isolate Bp 2723 through quantitative RT-PCR.

Gene	Primer sequence	Gene	Primer sequence
*bvgA*	F: *5*′*AGACCGTCAGCACCTACA*	*prn*	F: *5*′*TGTTCCGCATGAATGTCTTC*
	R: *5*′*GAGGTCTATCAGTTCCACCA*		R: *5*′*TGTTGGCAAGGGTAAAGGTC*
*bvgS*	F: *5*′*ATTACGTCAACCGCTACTTC*	*fim2*	F: *5*′*GCCGCAGTTCCGGATAAA*
	R: *5*′*GTTCAGGATGGACATCAGTT*		R: *5*′*CGTTTGGGTCGACTCGTTG*
*vag8*	F: *5′GGTTCACTGGTAGAGAGCAC*	*fim3*	F: *5′GGTGCGGGAAGCTGTAGTTC*
	R: *5*′*GTTGAGCAGGGACACATTAC*		R: *5*′*CGTAGTGGTGGTTGATGCTGT*
*bsp22*	F: *5*′*GAACTCGAAAGTGCCTACAC*	*ompQ*	F: 5′*CAACCAGCCTTTATGCCTATG*
	R: *5*′*ATGTCCATCTGTTGCGTATT*		R: 5′*GTCATTCCCACGCCAAAC*
*brkA*	F*: 5*′*GACGCAGGAGTTCAAAAG*	*bipA*	F: 5′*GACAGCGGTTTCTACCTGGA*
	R*: 5*′*TACGAAGCATAGAGGTTGTG*		R: 5′*CGCCACCTTGAAGTCATTCT*
*bcrH2*	F*:5*′*CTATGCCTGCAGAAGACC*	*ptxS*	F: 5′*TGTTCCGCATGAATGTCTTC*
	R*: 5*′*GAATCTGGATAGAGCGTGAG*		R: 5′*GACAGCGGTTTCTACCTGGA*
*fhaB*	F: *5*′*GCCACGATTTCACGGTGCA*	*16S*	F: 5′*TCAGCATGTCGCGGTGAAT*
	R*: 5*′*CAGCGTCGCGTCATGCT*		R: 5′*TGTGACGGGCGGTGTGTA*


### DNA Sequencing and Data Analysis

The *bvgS* gene was sequenced for all clinical isolates used in this study. In addition, the following genes and their promoter regions were sequenced for clinical isolate Bp2723: *fhaB*, *ptxS1*, *fim3*, *prn*, *bsp22*, *bcrH2*, *vag8*, *brkA*, and *bvgA* using the primers listed in **Table 2**. For chromosomal PCR amplification the procedure described by Van Loo and Mooi (2002) was employed. Briefly, 1 μL of DNA was added to 19 μL of buffer comprising 50% Hotstar Taq Master mix kit (Qiagen), 1 μM concentration of each primer, and 5–10% dimethyl sulfoxide. Amplification of genes was performed in a Hybaid Omnigene incubator. The PCR fragments were purified with QIAquick PCR purification kit (Qiagen) and sequenced with the primers used for amplification and internal primers (not shown). Sequence reactions were performed with an ABI Prism Big Dye terminator reaction Kit, and the reactions were analyzed using a 377 or 3700 ABI DNA Sequencer (Perkin–Elmer, Applied Biosystems). The resulting sequences were searched against NCBI nucleotide or non-redundant protein database by using BLAST tool.

## Results

### Planktonic Growth and Biofilm Formation Capacity on Abiotic Surfaces by *B. pertussis* Tohama I Strain and Clinical Isolates

**FIGURE 1:**
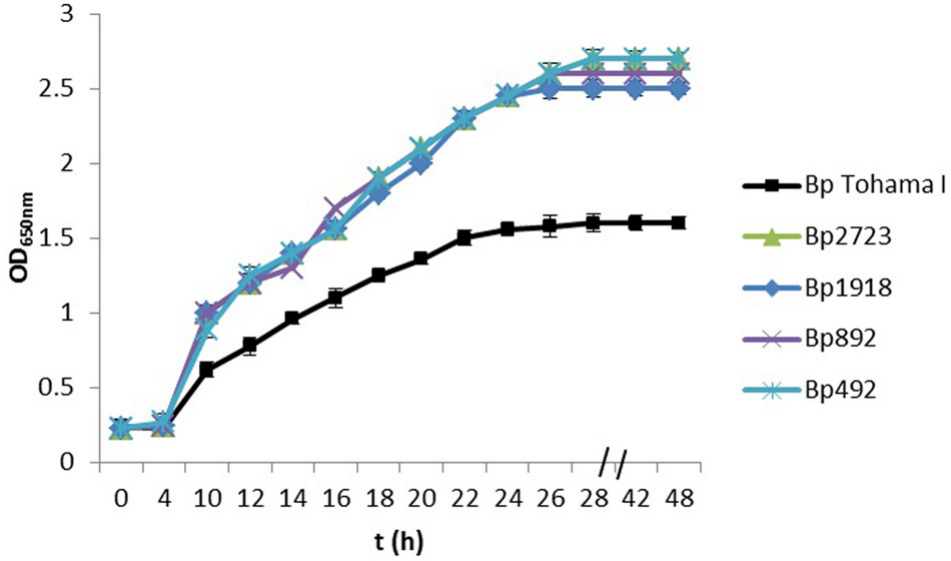
**Growth kinetics of *Bordetella pertussis* Tohama I strain and four clinical isolates in Stainer–Scholte (SS) liquid medium.** *B. pertussis* clinical isolates and *B. pertussis* Tohama I strain were grown planktonically for 48 h in SS broth. The biomass of clinical isolates after 30 h of culture was almost 70% higher than produced by the reference strain. Results are represented as the mean of values obtained from three independent experiments. The error bars indicate the standard deviations.

**FIGURE 2:**
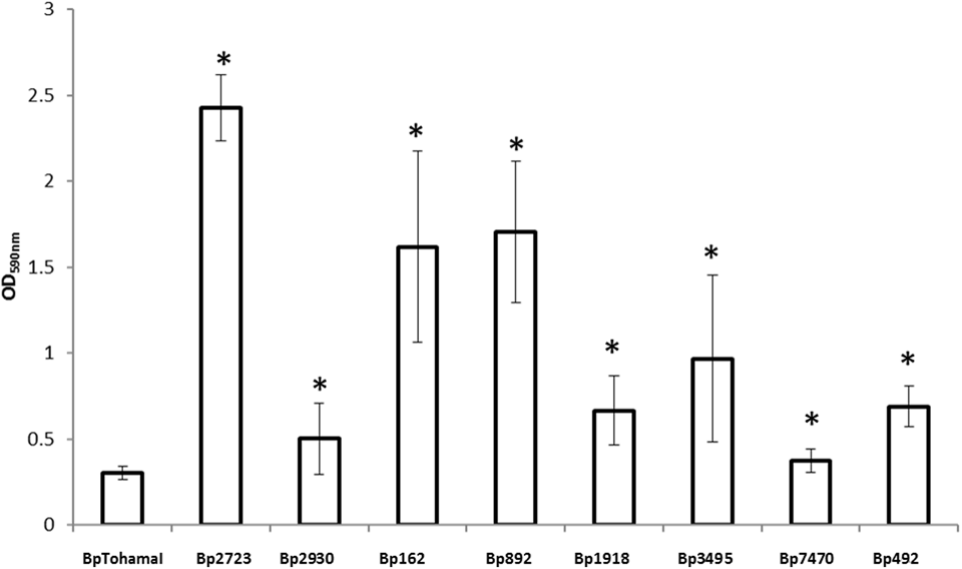
**Biofilm formation by *B. pertussis* Tohama I strain and clinical isolates on an abiotic surface.** Adhesion and biofilm formation capacity of *B. pertussis* Tohama I reference strain and eight clinical isolates after 72 h of growth (mature biofilm). Biofilm biomass was quantified by staining with crystal violet and absorbance was measurement at OD 590 nm. Each value represents the mean from three independent experiments and the bars indicate standard deviation. Statistically significant differences in Student’s test between each clinical isolate and Bp Tohama I biomass absorbance are indicated by symbols when present (^∗^*p* < 0.05).

**FIGURE 3:**
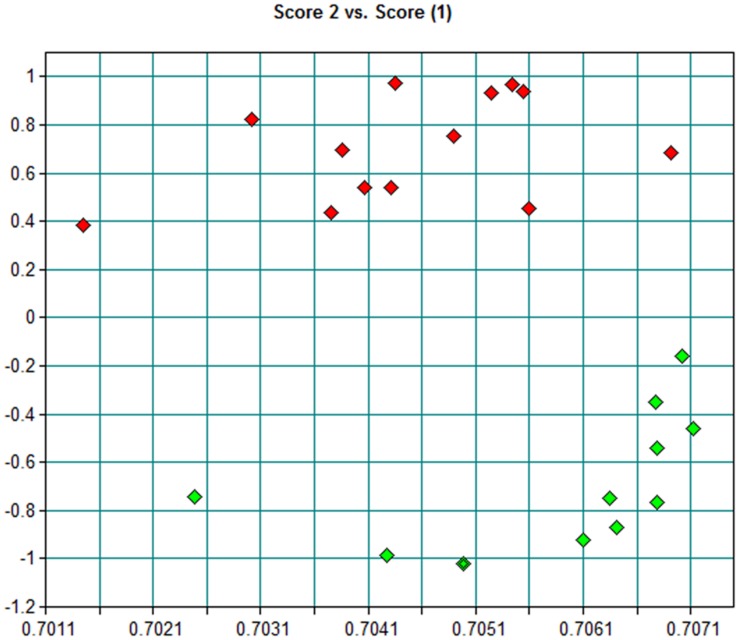
**Principal component analysis (PCA) scores scatter plots of biofilms FT-IR spectra from *B. pertussis* Tohama I strain (green) and *B. pertussis* 2723 isolate (red) in the 4000–650 cm^-1^ region**.

Using a collection of eight clinical isolates recovered in Argentina from whooping cough patients and the reference strain *B. pertussis* Tohama I, a comparative growth analysis under planktonic conditions in SS broth was performed. **Figure 1** shows the growth kinetics for Tohama I strain and the clinical isolates *B. pertussi*s 2723 (Bp 2723), *B. pertussis* 892, *B. pertussis* 1918, and *B. pertussis* 492. The four isolates are depicted as representative for the clinical isolates, which showed similar growth behavior (data not shown). At stationary phase the planktonic biomass of the isolates -measured by optical density- was approximately 70% higher than the biomass reached by *B. pertussis* Tohama I strain. From batch cultures, specific growth rate (μ) for each strain was calculated. *B. pertussis* 2723, as well as the other clinical isolates, exhibited similar specific growth rates of 0.091 ± 0.003 h^-1^ while the μ of Tohama I strain under the same experimental conditions was significantly lower (0.052 ± 0.002 h^-1^). Next, we compared the adhesion and the mature biofilm biomass of the clinical isolates on abiotic surface. All clinical isolates showed higher adhesion to polypropylene beads after 4 h of static incubation (data not shown) and higher biofilm biomass production after 72 h of culture (mature biofilm) compared to the reference strain (**Figure 2**). However, the final sessile biomass was different for each isolate. The *B. pertussis* 2723 strain was selected for further analysis since it exhibited fivefold more biofilm biomass as well as 70% more biomass under planktonic conditions compared to the reference strain Tohama I. The growth kinetics of this clinical isolate and *B. pertussis* Tohama I strain growing as biofilm were also studied. The clinical isolate Bp2723 showed a final biomass of 2.6 × 10^10^ CFU/cm^2^ while the biomass for reference strain was 2.5 × 10^9^ CFU/cm^2^. In addition, the specific growth rate for Bp2723 was 0.033 ± 0.002 h^-1^ and for Tohama I strain 0.028 ± 0.001 h^-1^. Next, and to differentiate the structure of mature biofilms produced by both Bp2723 clinical isolate and Tohama I strain, micrograph images of biofilms were obtained from CLSM stacks (**Supplementary Figure S1**). The images were then analyzed by using COMSTAT software. The images obtained showed characteristic biofilm architecture with channels for both, Bp Tohama I and Bp2723. A more profound analysis revealed that the clinical strain produced a bigger biofilm, characterized by a maximum thickness of around the double of that achieved by the reference strain. Five architectural parameters calculated for the two biofilms are provided in **Table 3**. These parameters show not only significant differences in the thickness of the biofilms but also in the covered surface and the roughness coefficient that were higher for the clinical strain biofilm. This analysis revealed an apparent higher complexity of the biofilm produced by Bp2723 compared to the biofilm produced by the reference strain and confirm its enhanced biomass production under this culture condition. To gain insight into the nature of the biofilm developed by the clinical isolate we employed FT-IR spectroscopy for the comparison of the chemical composition of the biofilms developed by the reference strain and the clinical isolate growing in similar environmental conditions. FT-IR spectroscopy, one of the most frequently used spectroscopic techniques to compare biochemical composition among biological samples, in association with PCA showed clear chemical differences between the FT-IR spectra obtained from both biofilms communities (**Figure 3**). Differences in biomass composition were detected between spectral areas assigned to protein and carbohydrates. The protein:carbohydrate ratio was 1.684 for Bp2723 and 1.142 for *B. pertussis* Tohama I strain, respectively. Based on this information we decided to explore the molecular basis for these significant phenotypic differences between the reference strain and the clinical isolate selected.

**Table 3:** Biometric parameters obtained from 72 h biofilms formed by *Bordetella pertussis* wild type (Bp Tohama I) and the clinical isolate *B. pertussis* 2723.

Quantitative parameters	Bp Tohama I	Bp2723
Thickness (μm)	10.33 (0.10)	14.55 (0.38)
Maximum thickness (μm)	17.78 (0.005)	36.56 (0.57)
Roughness coefficient	0.18 (0.005)	0.36 (0.07)
Covered surface	0.78 (0.03)	0.94 (0.04)
Surface to biovolume ratio (μm^2^/μm^3^)	1.72 (0.008)	1.93 (0.42)


*The features of the biofilms were quantified using the program COMSTAT. Parameters correspond to mean values. Standard errors are indicated.*

### 2D-DIGE Analysis and Protein Identification

**FIGURE 4:**
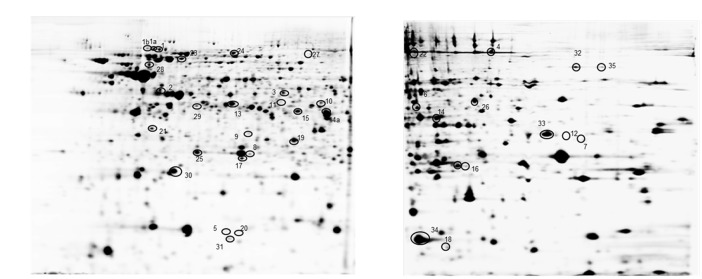
**Representative image of a 2D gel of *B. pertussis* at pH 4–7 **(A)** and pH 6–9 **(B)**.** Circled spots correspond to at least threefold differentially expressed cytosolic proteins in *B. pertussis* 2723 compared to Tohama I growing under planktonic and biofilm conditions (see **Supplementary Table S1** for details).

*Bordetella pertussis* Tohama I strain and the clinical isolate *B. pertussis* 2723 were grown in parallel as planktonic cells for 24 h in SS medium (exponential phase) and as biofilm on polypropylene beads for 72 h (mature stage). To investigate the differentially expressed proteins in the reference strain and the clinical isolate under both culture conditions, a comparative proteomic analysis was performed. The soluble cellular protein fraction was isolated from three replicates per strain and growth condition, and subjected to differential 2D DIGE analysis in two pH ranges (4–7 and 6–9) and a PCA was carried out. As shown in **Supplementary Figure S2**, the statistical analysis of each biological replicate, clearly indicates a distinct clustering of the four groups demonstrating a high reproducibility between the replicate samples. In addition, the analysis demonstrates that the highest variation was found to be strain-dependent (PC1), whereas PC2 discriminates the different growth conditions. Representative 2D electrophoresis patterns of bacterial proteins based on the internal standard sample for both pH ranges are depicted in **Figure 4**. The global proteome analysis showed that out of a total of 1275 spots analyzed, 65 proteins (5.1%) were differentially expressed in *B. pertussis* 2723 compared to the reference strain growing attached to surface and under planktonic culture conditions. Forty eight differentially expressed protein spots were selected for protein identification based on a combination of selection criteria as published elsewhere (Radwan et al., 2008). MALDI–ToF–MS–MS analysis resulted in the identification of 35 different proteins and/or protein species (**Supplementary Table S1**). The clinical isolate showed, in comparison to Tohama I strain 10 up-regulated proteins (*p* < 0.05) and five down-regulated proteins (*p* < 0.05) growing under biofilm conditions, and 27 proteins up-regulated and eight proteins down-regulated growing under planktonic conditions. These differentially expressed proteins can be assigned to five functional categories, namely metabolism-energy production, amino acid and protein synthesis, transport, virulence, and cellular process (**Supplementary Table S1**). More specifically, within the metabolic group, four proteins (aconitate hydratase –*acnB*-, dihydrolipoamide succinyl transferase component of 2-oxoglutarate dehydrogenase complex –*odhB*-, citrate synthase –*gltA*- and enoyl-CoA hydratase/isomerase –*acnA*-), related to the energy production were found in higher abundance in the clinical isolate under both culture conditions. Three out of four proteins belonging to “amino acids and protein biosynthesis” pathways were found down regulated in the clinical isolate 2723. These proteins correspond to enzymes involved in phenylalanine, tyrosine, tryptophan, and lysine biosynthesis. The fourth protein, cystathionine beta-lyase -*metC*-, which is related to methionine synthesis and sulfur metabolism, was up-regulated in *B. pertussis* 2723. In addition, three proteins involved in amino acids transport [leu/ile/val (branched chain amino acid-) –*Q7VYN1*- binding protein –*livJ*-, amino acid- binding periplasmic protein –*Q7VS83*-, ABC transporter ATP binding protein –*Q7VTG4-*] and four proteins related to stress response and adaptation (putative Zinc protease –*Q7VVY4*-, antioxidant protein –*Q7VZE7*-, chaperone protein DnaJ –*Q7VVY3*-, and protein tex –*Q45388*-) were found to be up-regulated in the clinical isolate under both culture conditions (**Supplementary Table S1**). Noteworthy, four BvgAS-activated virulence factors, BcrH2, OmpQ, Vag8, and BrkA, were found under both growth conditions in higher abundance in the clinical isolate 2723 than in the reference strain. One of these proteins, namely BcrH2, is a chaperone protein member of the Type III secretion system (T3SS). T3SS correspond to an injection system that delivers virulence factors into the host cells changing its physiological functions (Mattoo et al., 2004; Medhekar et al., 2009). It is known to protrude from bacterial outer membrane of many Gram-negative bacteria. Another BvgAS regulated protein found to be differentially expressed in the clinical isolate is OmpQ, an outer membrane porin protein that so far has no clear assigned function (Finn et al., 1995). In addition, Vag8 and BrkA were also found in higher abundance in the clinical isolate. Vag8 is an autotransporter recently described to participate in serum resistance and described to bind C1; and BrkA, another serum resistance protein of *B. pertussis*, was reported as necessary for efficient colonization of mice (Finn and Amsbaugh, 1998; Barnes and Weiss, 2001; Marr et al., 2011). These latter observations were particularly interesting since we detected four BvgAS-activated proteins highly expressed in the clinical isolate compared to the Tohama I strain in soluble fraction. As BvgAS-activated genes play an essential role in biofilm development, we next studied the expression of BvgAS regulated adhesins in both strains under biofilm and planktonic culture conditions.

### qRT-PCR for *bvgAS* and its Positively Regulated Genes

**FIGURE 5:**
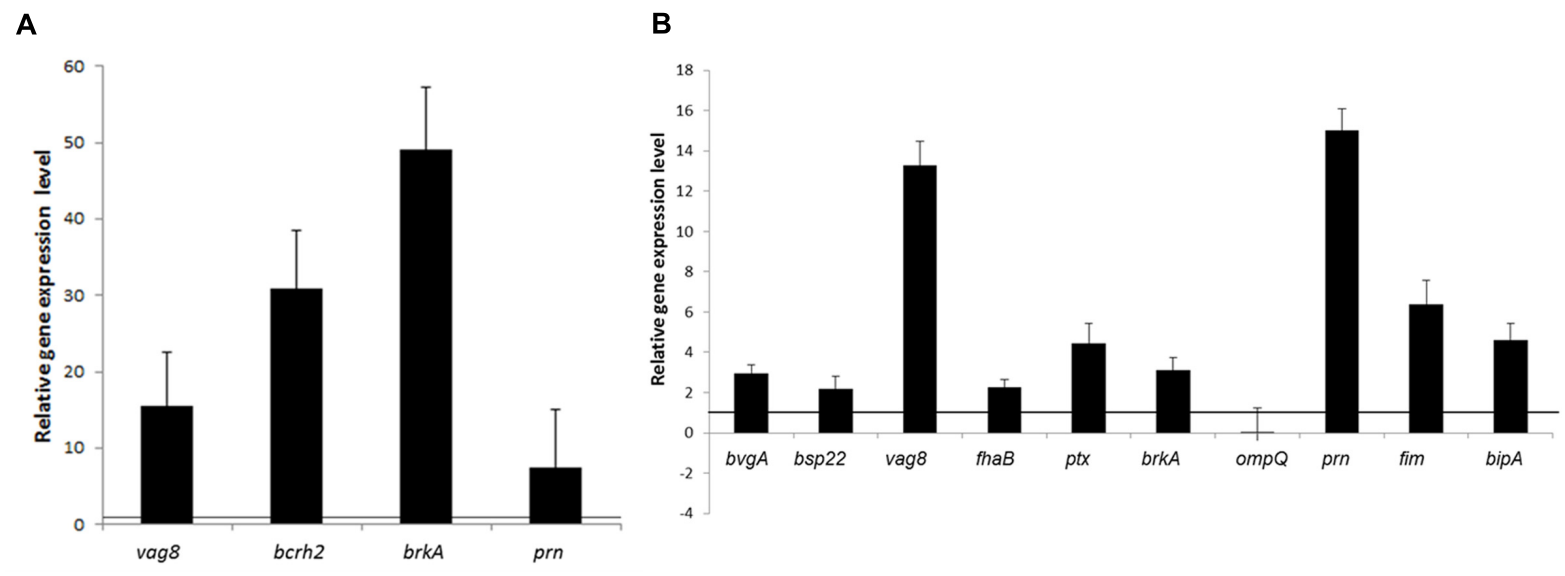
**Relative transcription levels of virulence genes.** Transcription levels of selected genes positively regulated by the BvgAS system were quantified by qRT-PCR. Transcription levels from the clinical isolate *B. pertussis* 2723 are shown relative to those from the Tohama I strain growing in planktonic **(A)** and biofilm culture **(B)**. The black line indicates equal relative gene expression level, lower values indicate low expression in the clinical strain Bp2723 and higher value indicates up regulation of the genes. In the case of *fim* genes, expression of *fim2* gene in Tohama I was compared to the corresponding *fim 3* of the clinical isolate.

qRT-PCR was performed to measure mRNA expression levels of BvgAS-regulated genes: *fhaB*, *fim* (*fim2* for Tohama I and *fim3* for Bp2723), *prn*, and *bipA* in cells grown under biofilm and planktonic conditions. In addition *vag8*, *brkA*, *bcrH2*, and *ompQ* genes, encoding the four proteins which showed increased levels of expression by *B. pertussis* 2723 compared to Tohama I strain in the cytosolic proteome (**Supplementary Table S1**), as well as *bvgS, bvgA*, and *ptx* genes were included in the analysis. This approach revealed a higher mRNA expression levels of *vag8*, *bcrH2, prn*, and *brkA* in planktonic cells of the clinical isolate compared to Tohama I strain (**Figure 5A**). Interestingly, significantly higher mRNA expression levels of *fhaB*, *fim3*, *ptxS1*, *bipA, vag8*, *prn*, *bvgA, bsp22*, and *brkA*, genes were found in sessile cells of the clinical isolate compared to the reference strain (*p* < 0.05; **Figure 5B**). The higher expression of *fhaB, prn*, *fim3*, and *bipA* genes, might probably be associated with increased biofilm formation capacity depicted by the clinical isolate, since these genes encode adhesins reported to participate in the development of *B. pertussis* mature biofilm (Serra et al., 2011; Sugisaki et al., 2013; de Gouw et al., 2014).

### Sequence Analysis of *bvgA*, *bvgS*, Virulence Genes, and Promoters

**FIGURE 6:**
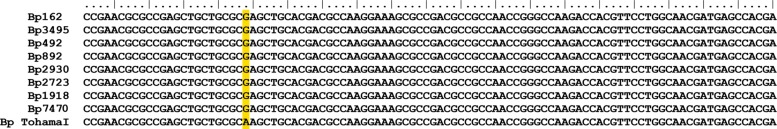
**Comparative sequence analysis of *bvgS* gene.** The *bvgS-*PAS domain sequence for *B. pertussis* Tohama I strain and the eight *B. pertussis* clinical isolates analyzed are shown. The single nucleotide mutation at position 2113 is highlighted in yellow.

To determine whether specific nucleotide changes within virulence genes, or their promoter regions had occurred in Bp2723, the respective DNA sequences and their flanking regions were analyzed. No changes were detected when compared with the published sequences of Tohama I strain (GenBank accession number BX470248.1). However, one nucleotide polymorphism, which corresponds to a single exchange of an A for a G at position 2113 of the *bvgS* gene (**Figure 6**), resulting in the replacement of K by E in the amino acid sequence of BvgS protein was observed for Bp 2723. Based on this result, we sequenced the *bvgS* gene of all clinical isolates used in this study and surprisingly found the same mutation in each clinical isolate (**Figure 6**). Interestingly, the same nucleotide exchange has been previously described by Herrou et al. (2009) for strains circulating in the Netherlands.

### Effect of E at 705 Position of BvgS Sensor on the Biofilm Formation Ability of *B. pertussis*

**FIGURE 7:**
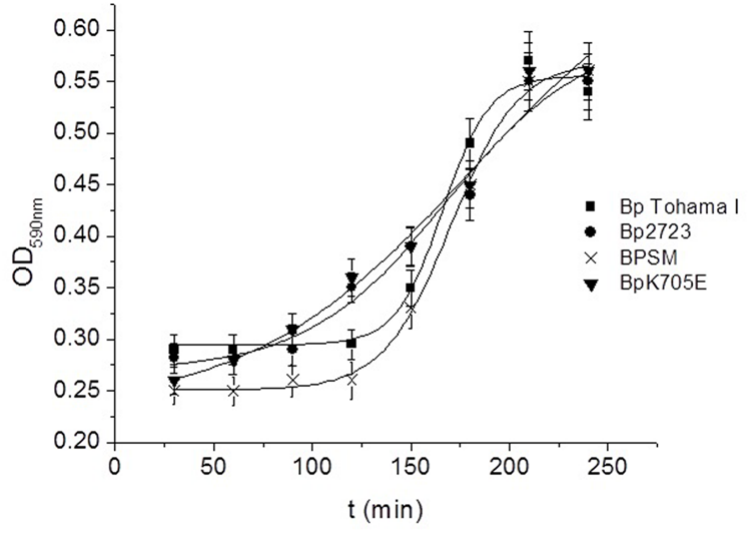
**Adhesion kinetic of modulated *B. pertussis* to polypropylene beads.** *B. pertussis* Tohama I strain (square spots), Bp 2723 (circle spots), the mutant strain for the BvgS, BpK705E (horizontal triangles) and its wild type strain, BPSM (vertical triangles) were modulated in agar plates and planktonic cultures under 40 mM MgSO_4_. Then the cells were harvested and suspended in non-modulating media to perform the adhesion assay to polypropylene beads in static conditions. Every 30 min. a sample was taken to measure the adhered biomass to beads using the violet crystal staining.

To evaluate whether the mutation detected in the *bvgS* gene could affect biofilm formation ability, *B. pertussis* 2723 and *B. pertussis* Tohama I strain were tested for their capacity for biofilm development. Growth was evaluated in tube-bioreactors containing polypropylene beads. The mutant strain Bp_K705E_ with an E at position 705 of BvgS and the wild type strain (BPSM) with a K at position 705 were also included in this analysis. Our results did not reveal any significant differences in the mature biofilm biomass of Bp_K705E_ strain, the wild type BPSM and Tohama I strain after 72 h of culture (data not shown), suggesting that this point mutation in the *bvgS* gene is not associated to the increased biofilm biomass shown by the clinical isolate. Since this mutation has been previously described to confer a sensitive response to modulatory agents, such as MgSO_4_ and nicotinic acid, we investigated whether this mutation could trigger a faster attachment of bacteria when they are transferred from a modulatory environment to a non-modulatory one. The four strains described above were therefore modulated using SS culture medium with the addition of 40 mM MgSO_4_ and then incubated in a non-modulating SS medium under static conditions with polypropylene beads for 4 h. Under this condition, the surface adhesion of each strain was monitored every 30 min. Noteworthy, the clinical isolate Bp2723 and the mutant Bp_K705E_ strain adhered to polypropylene beads significantly faster than Tohama I and BPSM wild type strains (**Figure 7**). The kinetic adhesion of the Tohama I and BPSM wild type strains showed a lag period of 120 min while an increase of biomass attached was already observed for the clinical isolate and Bp_K705E_ strain after 30 min. These findings suggest that the *bvgS* allele coding for the 705 E protein variant might be associated with an accelerated expression of adhesins involved in the initial adhesion steps.

## Discussion

In the current work, we investigated the biofilm formation capacity of eight argentinean *B. pertussis* clinical isolates recovered over a seven years time period in a local children hospital compared to a laboratory adapted strain grown under biofilm conditions. Clinical strains showed an increased ability to grow attached to polypropylene surfaces compared to the laboratory strain. The architecture of biofilm developed by the reference strain was compared with the one developed by the isolate Bp2723 growing in similar culture conditions by using CLSM. We found a marked variability in three dimensional biofilm structures between the two strains studied. The measurement of parameters extracted from confocal stack images analyzed by COMSTAT software such as thickness, roughness coefficient, surface to biovolume ratio, revealed that the biofilm developed by clinical isolate is significantly thicker than the formed by the reference strain. In addition to the differences between the architectures of both biofilms a FT-IR spectroscopic analysis also showed clearly phenotypic variations between these two biofilms. Thus, to better understand the different growth performances between clinical isolates and Tohama I strain we carried out a proteome investigation. The major functional groups of differentially expressed proteins in the clinical isolate included energy metabolism, transport, stress and regulation, and virulence factors. Seven proteins involved in metabolism and energy production were found up-regulated in sessile cells of the clinical isolate *B. pertussis* 2723. Among them the citrate synthase that catalyzes the first reaction in the TCA cycle represents an important control point for determining the metabolic rate of the cell (Park et al., 1994). The higher expression of proteins associated to energy production under respiratory conditions was attributed primarily to the cell biosynthetic needs to produce higher biomass quantities. Our results indicate that the clinical isolate 2723 could have different metabolic and energetic requirements than the reference strain, which is supported by the higher final biomass reached for this isolate both in liquid medium and biofilm growth conditions. However, this higher capacity of biofilm formation is not associated with a higher growth rate. The TCA cycle is not only a central point in the metabolism of living organisms but also important for the survival of infectious biofilms. Therefore, its inhibition could be a promising strategy for the control of biofilms (Yahya et al., 2014). Similar results were previously reported in comparative proteomic studies of two *Burkholderia cenocepacia* isolates retrieved from a chronically infected cystic fibrosis patient. *B. cenocepacia* isolate obtained after 3 years of persistent infection and antibiotic therapy, showed an up-regulation of citrate synthase (Madeira et al., 2011), which was reported to be important for biofilm formation and virulence (Subramoni et al., 2011). The results from the *B. cenocepacia* study as well as the results from our current study on *B. pertussis*, point toward a tight link between primary metabolism and biofilm formation capability.

Our proteome analysis revealed an increased expression of the Bvg-activated factors BcrH2, OmpQ, BrkA, and Vag8 in the clinical isolate *B. pertussis* 2723. These proteins are positively regulated by the *B. pertussis* BvgAS two-component signal transduction system. This system is known for its key role in the regulation of *Bordetella* virulence gene expression including adhesins and toxins, and it has also been shown to be determinant for the ability of *Bordetella* species to produce biofilms (Irie et al., 2004; Mishra et al., 2005; Serra et al., 2007). If BvgAS is not active *B. pertussis* is unable to adhere to respiratory tract and colonize the host (Bassinet et al., 2000; Scheller et al., 2015). Using quantitative real-time PCR assays we analyzed the relative expression level of adhesin genes, known to be positively regulated by the BvgAS system. After 72 h of biofilm growth these genes were transcribed at higher levels in the clinical isolated compared to Tohama I strain. In addition the *bvgA* regulatory gene showed three times higher transcription in *B. pertussis* 2723 sessile cells compared to reference strain, although no significant increase was found on the protein level. The latter results underpin the importance and value of combinatory analysis on transcriptional and translation levels. Although beyond the scope of our current work, these studies should be expanded in the future to post-translation and metabolic level to gain a holistic picture of pathogen host adaptation mechanisms.

The adhesins tested, namely FHA, Fim3, Prn, and BipA, showed higher transcriptional levels by qRT-PCR in the clinical isolate grown as biofilm compared to the reference strain Tohama I. FHA, one of the main adhesins described for *B. pertussis*, is involved in different steps of biofilm formation *in vitro* and *in vivo* in mouse nasopharynx, contributing not only to the first adhesion to the surface but also enhancing cell–cell interactions (Serra et al., 2011). Recently, we also reported that BipA is a common signature of *B.* pertussis biofilms (de Gouw et al., 2014). Interestingly, although Prn negative strains are now increasingly being isolated from patients with whooping cough (Barkoff et al., 2012; Pawloski et al., 2014), our results showed that this protein is up-regulated in biofilm culture for *B. pertussis* 2723. Proteomics and targeted transcriptomics approaches provide a picture of the changes between reference strain and a clinical *B. pertussis* isolate growing in similar culture conditions. These results are in agreement with the ones from FT-IR analysis, which show a chemical abundance of proteins in the mature structure of the biofilm produced by the clinical isolate. Therefore, it is tempting to speculate that: (i) the high expression of adhesins, mediating a faster and enhanced attachment, as well as (ii) the higher expression of enzymes involved in energy metabolism, leading to the augmented biomass, are responsible for the robust biofilm structure of the clinical isolate. When bacteria are under stress conditions, they often get together to form biofilms, which suggests that this bacterial lifestyle increases the fitness of the cells in harsh environments. Differential gene expression patterns between Tohama I strain and clinical isolates planktonic cells were previously attributed to either sequence divergence in *cis*-regulatory regions or variation in the levels, activity, or encoding of transcriptional regulatory proteins (Cummings et al., 2006). However, in our current study, the higher transcription of adhesin genes could not be assigned to specific polymorphisms in the sequences of structural genes or promoters, suggesting that trans-acting factors could be involved. A single nucleotide mutation was found in *bvgS* gene of all clinical isolates tested, resulting in an exchange of lysine by glutamic acid at position 705 in the linker domain of the sensor protein. Interestingly, Herrou et al. (2009) reported the same mutation previously. Using an experimental infection model, they demonstrated that this mutation in the BvgS sensor of *B. pertussis* BPSM strain does not lead to a better pulmonary survival of the pathogen, though a faster response to modulatory agents like MgSO_4_ and nicotinic acid was observed. The latter results are in agreement with our current findings. After being modulated by MgSO_4,_ the clinical isolate Bp2723 were transferred to SS medium and incubated under non-modulating conditions with polypropylene beads. Under the latter culture condition cells showed faster adherence to abiotic surfaces than the reference strain. In our experimental conditions, both, the clinical isolate Bp2723 and the Bp_K075E_ strain showed an accelerated adhesion kinetic to polypropylene beads compared to Tohama I and the wild type BPSM strain. This high phase variation capability might represent an important adaptive advantage during pathogen colonization of its host. Fast adhesion suggests that the phases are tuned to different environmental niches favoring spatially defined regulation. This timing for attachment could promote persistence by protecting bacteria from the clearance occasioned by hydrodynamic forces in upper respiratory tract and the killing activity of host defense mechanisms. It is important to note that the increase of adhered cells (Bp2723 and Bp_K075E_ strain) detected after 30 min of incubation under our experimental conditions is not due to growth on the surface since these sessile bacteria have a reduced specific growth rate (μ = 0.033 h^-1^, and duplication time of 21.0 h). *B. pertussis* transmission from human to human can probably occur by aerosolized respiratory droplets containing modulated bacteria. Thus, the results shown in our work could be important for the understanding of the rapid adaptation of clinical isolates to new environment. Our combinatory approach of proteomics, targeted transcriptional and genetic analysis revealed that multiple realms of regulation are governing the adaptation of *B. pertussis* to biofilm lifestyle.

## Conclusion

The divergent biofilm responses between *B. pertussis* clinical isolates and the laboratory adapted reference strain suggest that clinical isolates probably evolved in order to increase their potential and capacity to form biofilm and eventually to adapt rapidly to the fluctuations that they encounter at the site of infection. To date the emergence of pertussis remains a critical issue that should gear researchers to develop novel control measures by considering particularly the biofilm as a *B. pertussis* way of life.

## Author Contributions

Conceived and designed the experiments: OY and ME-S. The experiments were performed by LA, TG, NC, DdG, MV, DS. Analysis of data: all the authors. Contributed reagents/materials/analysis tools: OY, ME-S, and FM. Contributed to the writing of manuscript: all the authors.

## Conflict of Interest Statement

The authors declare that the research was conducted in the absence of any commercial or financial relationships that could be construed as a potential conflict of interest.
