# Supplementary material for: Bordetella pertussis Isolates from Argentinean Whooping Cough Patients Display Enhanced Biofilm Formation Capacity Compared to Tohama I Reference Strain
Source: Front Microbiol. 2015 Dec 8;6:1352. doi: 10.3389/fmicb.2015.01352 (PMC4672677; doi:10.3389/fmicb.2015.01352)
Supplement: Supplementary file 1 [file Image_1.PDF]

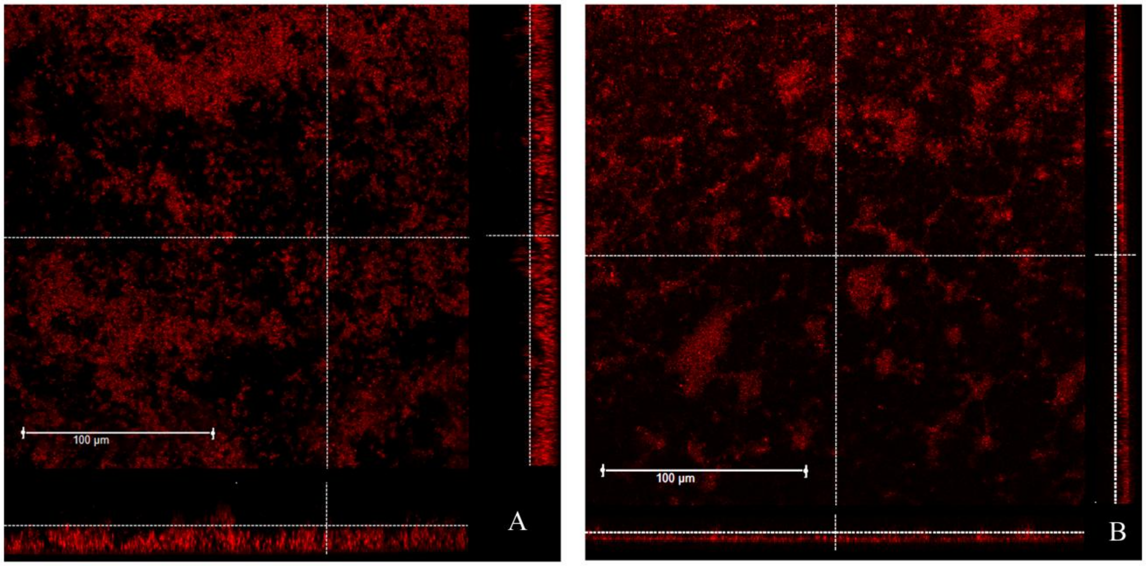

Image S1. CLSM biofilm architecture of *B. pertussis* 2723 clinical isolate (left column, A) and *B. pertussis* Tohama I strain (right column. B).
