# Supplementary material for: Bordetella pertussis Isolates from Argentinean Whooping Cough Patients Display Enhanced Biofilm Formation Capacity Compared to Tohama I Reference Strain
Source: Front Microbiol. 2015 Dec 8;6:1352. doi: 10.3389/fmicb.2015.01352 (PMC4672677; doi:10.3389/fmicb.2015.01352)
Supplement: Supplementary file 2 [file Image_2.PDF]

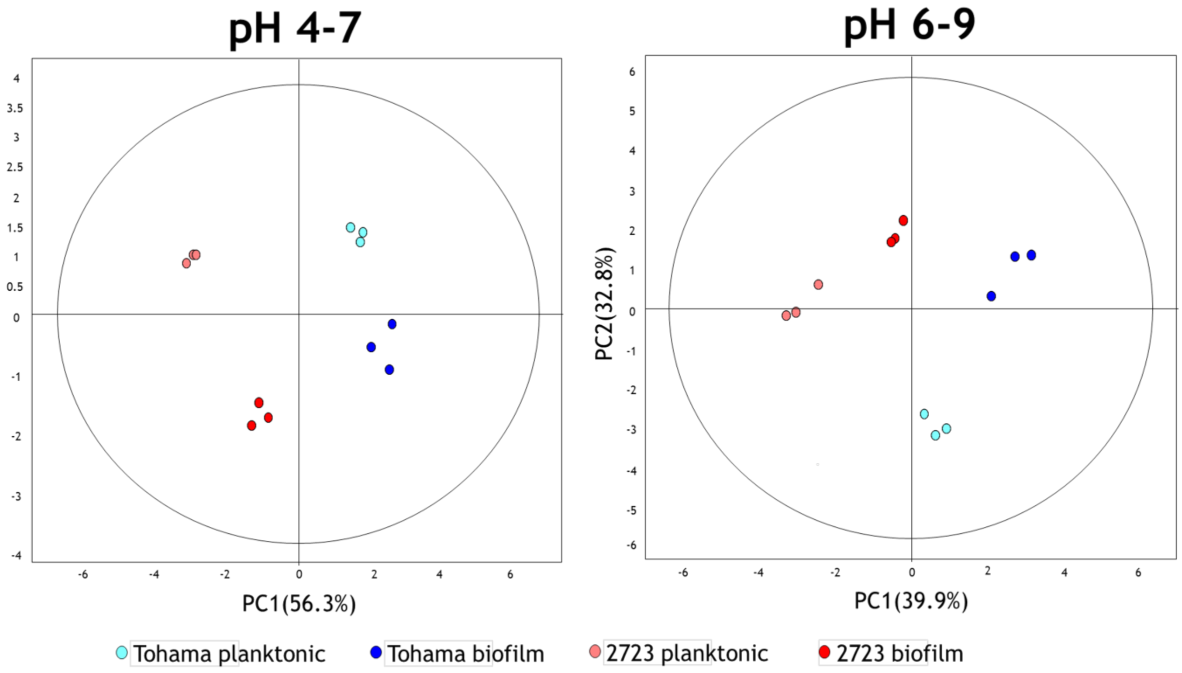

Image S2. Principal Component analysis. Each data point refers to one replicate of an experimental group based on the global expression values for all differentially expressed spots (1-way ANOVA; $p<0.05$), as its possible to observe, the main differences in protein expression are expressed between strains and in a second place between the culture conditions.
