# Supplementary material for: Bordetella pertussis Isolates from Argentinean Whooping Cough Patients Display Enhanced Biofilm Formation Capacity Compared to Tohama I Reference Strain
Source: Front Microbiol. 2015 Dec 8;6:1352. doi: 10.3389/fmicb.2015.01352 (PMC4672677; doi:10.3389/fmicb.2015.01352)

**Table S1.** Differentially expressed proteins in *B. pertussis* 2723 in relation to *B. pertussis* Tohama I strain, grown under planktonic and biofilm conditions. The proteins were identified by MALDI-ToF/MS.


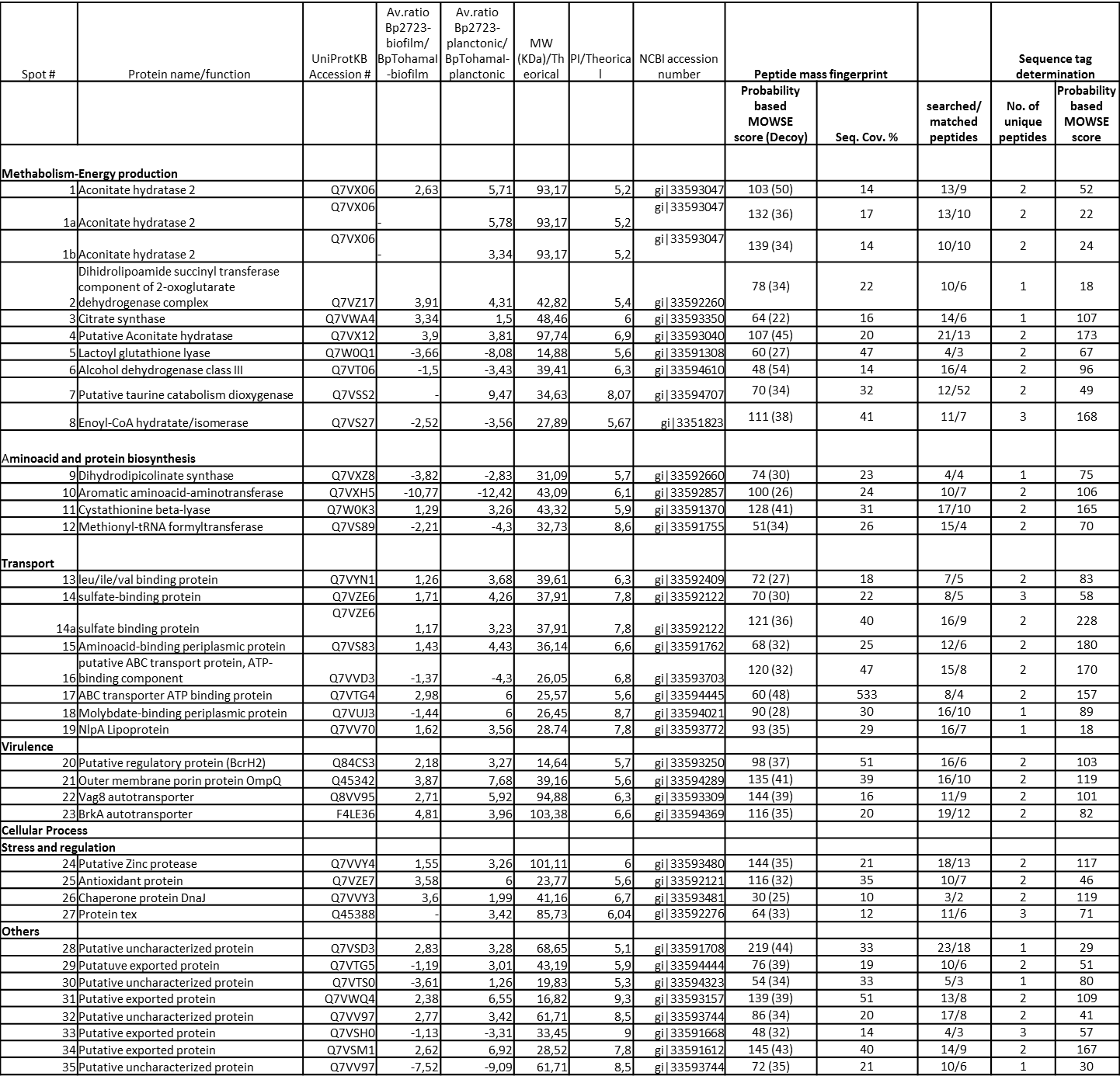

Supplement: Supplementary file 3 [file Table_1.DOCX]
